# The association of posterior reversible encephalopathy syndrome with COVID-19: A systematic review

**DOI:** 10.1016/j.amsu.2021.103080

**Published:** 2021-11-20

**Authors:** Sadaf Iftikhar, Aqeeb Ur Rehman, Muhammad Zain Ameer, Ahmad Nawaz, Muhammad Aemaz Ur Rehman, Hareem Farooq, Abyaz Asmar, Muhammad Ebaad Ur Rehman

**Affiliations:** a Mayo Hospital, King Edward Medical University, Lahore, 54000, Pakistan; b King Edward Medical University, Lahore, 54000, Pakistan; c Allama Iqbal Medical College, Lahore, 54550, Pakistan

**Keywords:** Cerebral edema, COVID-19, Posterior leukoencephalopathy syndrome, Posterior reversible encephalopathy syndrome, PRES, SARS-CoV-2

## Abstract

The rise in Coronavirus disease 2019 (COVID-19) cases is revealing its unique neurological manifestations. In light of the emerging evidence, a possible association with Posterior Reversible Encephalopathy Syndrome (PRES) is being consistently reported. We conducted a systematic literature search on four databases namely Pubmed/MEDLINE, Cochrane, Google Scholar, and Science Direct. After rigorous screening as per Preferred Reporting Items for Systematic Reviews and Meta-Analyses (PRISMA) guidelines, a total of 34 articles describing 56 cases were selected as a part of this review. The mean age of the patients was 56.6 ± 15.3 years. The most common clinical presentation of PRES was altered mental status (58.9%) followed by seizures (46.4%) and visual disturbances (23.2%) while hypertension and diabetes mellitus were the most commonly reported comorbidities. 91.1% of the cases reported Magnetic Resonance Imaging (MRI) or Computed Tomography (CT) findings suggestive of PRES in the brain. Symptomatic management was employed in most of the cases to control seizures and blood pressure, and 44 patients (78.5%) fully or partially recovered. The most likely underlying mechanism involves COVID-19 mediated cytokine storm syndrome that leads to endothelial damage and increased permeability of the cerebral vessels, thus causing the characteristic edema of PRES. High neuronal and glial cell expression of Angiotensin Converting Enzyme-2 (ACE-2) receptors also suggests the possibility of direct viral damage. Since timely diagnosis and treatment reports a good prognosis, it is vital for physicians and neurologists to be well-versed with this association.

## Highlights

• Posterior Reversible Encephalopathy Syndrome (PRES) is a neurological complication of COVID-19.
• SARS-CoV-2 disrupts the integrity of cerebral blood vessels through cytokine storm, thus driving the edema of PRES.
• COVID-19 is probably additive with hypertension, diabetes and tocilizumab in causing PRES.
• Timely diagnosis and treatment are necessary to avoid complications such as cerebral hemorrhage, epilepsy and vision loss.
• A favorable outcome is observed in most cases.

## 1 Introduction

The Posterior Reversible Encephalopathy Syndrome (PRES) is a neurological complication that usually arises as part of a greater underlying disease, such as renal failure, autoimmune disorders, and acute hypertension (including eclampsia) [1]. A common pathophysiological theme that accompanies all of these disorders is the disruption of the blood-brain barrier secondary to endothelial dysfunction [2], resulting in cerebral edema in posterior parts of the brain. The parietal and occipital lobes are characteristically involved [3], with brainstem and spinal cord involvement reported only in atypical cases [4,5]. The common presenting complaints include seizures, headache, visual disturbances, and altered sensorium [6,7]. Diffusion-weighted MRI is the imaging modality of choice for confirming the presence of typical or atypical radiographic features [8,9].

As of October 18, 2021, more than 239 million cases of COVID-19 have been reported globally [10], and with no signs of the pandemic being brought under control in near future, especially in the developing world, it is imperative to understand its various manifestations. Among the many neurological manifestations of COVID-19, PRES has been consistently reported in literature from the inception of this virus to date. Mozhdehipanah et al. described the first case of PRES secondary to COVID-19 in May 2020 and since then, the steep rise in cases of PRES has led the medical fraternity to believe that an unrecognized association between SARS-CoV-2 and PRES exists. SARS CoV-2 is well known to cause endothelial dysfunction and damage through the “cytokine storm syndrome” [11], and hence qualifies as a plausible driver of the characteristic edema seen in PRES. The large emerging literature on this topic requires an integrated write-up to better understand the clinical presentation, pathophysiology, prognosis and outcome of this clinical association. An understanding of the disease process will allow timely diagnosis, and improve prognosis by preventing the development of any late-stage complications of PRES like intracranial hemorrhages and epilepsy [3,12]. This review serves to not only educate and guide the practicing physicians, but also provides a foundation for researchers to build on this work as we expand the scientific understanding of this disease in future. It is the first systematic review to provide a comprehensive outline of available evidence on COVID-19-related-PRES from both clinical and pathophysiological standpoint.

## 2 Methods

This article is fully compliant with the PRISMA (Preferred Reporting Items for Systematic Reviews) 2020 statement, and follows the criteria outlined in PRISMA [13]. This review has been registered with the Research Registry (UIN: reviewregistry1255).

### 2.1 Search strategy

A systematic literature search was conducted on the following four databases: Pubmed/MEDLINE, Cochrane, ScienceDirect and Google Scholar. The search string consisted of a combination of keywords and Mesh terms such as: COVID-19”[MeSH], “Covid*”, “SARS-CoV-2”, “Posterior reversible encephalopathy syndrome”, “Reversible posterior leukoencephalopathy syndrome”, “Reversible posterior cerebral edema syndrome”, “PRES”, "Posterior Leukoencephalopathy Syndrome"[Mesh] etc. The complete search string used in each database is provided in the supplementary files. No filter in terms of time, study design, language, country of publication etc. was used in order to retrieve all the available literature.

### 2.2 Study selection and data extraction

**Fig. 1:**
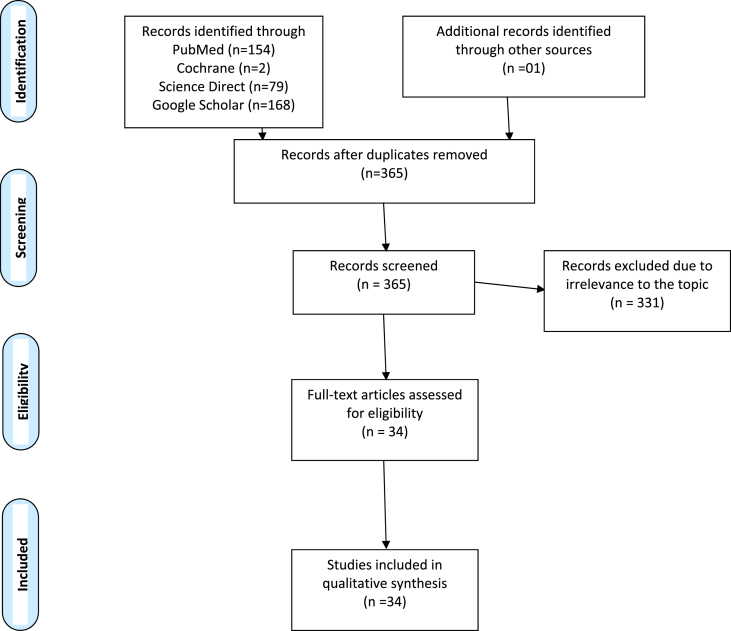
Prisma flow diagram.

The articles were selected and screened according to the PRISMA flowchart [Fig. 1]. The records identified through the preliminary search were downloaded into Mendeley and duplicates were removed. Two independent reviewers H.F and M.A.R performed the screening and concluded with a consensus that case reports, case series, and letters to the editor etc. reporting cases of PRES in COVID-19 have been published on this topic. The bibliographies of these cases were sieved to identify any missed cases. The data was curated in the form of two tables for COVID-19 associated PRES with one focusing on demographics, clinical features and outcomes while the other was based on diagnostic imaging and relevant investigations. Continuous variables were presented as means ± standard deviations and categorical variables were presented as absolute values and percentages. Microsoft Excel was used for data extraction and statistical analysis for this study. The references were added through Zotero.

### 2.3 Quality assessment

The quality of the included articles was assessed by Joanna Briggs Institute Critical Appraisal Tool [14]. Three reviewers (M.A, Z.A, A.N) first independently scored each article and then awarded a consensus score to each. The score report is provided in the supplementary files. The article has also been self-evaluated through the AMSTAR 2 checklist [15], which is available as a supplementary file. The level of compliance with AMSTAR 2 came out to be “moderate”. As only case reports were included in the analyses, we could not conduct a meta-analysis.

## 3 Results

Our search of the four databases identified 403 articles, while one was identified through miscellaneous sources. 39 articles were excluded due to duplication and 331 were removed due to irrelevance to the subject. The remaining 34 articles, including case reports, case series, letters to the editor, pictorial essays etc. reporting cases of PRES in COVID-19 were finally shortlisted after rigorous screening [[16], [17], [18], [19], [20], [21], [22], [23], [24], [25], [26], [27], [28], [29], [30], [31], [32], [33], [34], [35], [36], [37], [38], [39], [40], [41], [42], [43], [44], [45], [46], [47], [48], [49]]. Since a total of 31 patients were described in the nine case series, individual data of 56 patients is described in our systematic review in the form of two tables [Table 1 and Table 2].

**Table 1:** Demographics, presentation and outcome of PRES in COVID-19 patients.

Patient No.	Author, Year	Age (Years), Gender (M/F)	Comorbidities	Ventilator Status	Treatment given for COVID-19	Blood Pressure	PRES Symptoms	Risk Factors for PRES	PRES Management	Outcome
1	Lucia Princiotta Cariddi et al. (2020) [14]	64, F	HTN, GERD, Hyperuricemia, Dyslipidemia, OSA, Afib	(+)	Darunavir/Cobicistat, HCQ, Oxygen	High	AMS, Blurry Vision, Hypotonia, Reduced DTRs	HTN	–	Recovered
2	Pria Anand et al. (2020) [15]	61, F	–	(+)	Remdesivir, Anti-IL1	High	AMS	–	AEDs	Recovered
3	Pria Anand et al. (2020) [15]	52, F	HIV	(+)	None	High	Seizures, Gaze Deviation	–	–	Recovered
4	Amit Agarwal et al. (2020) [16]	27, F	–	–	–	–	AMS	–	–	Death
5	A.M. Francesch et al. (2020) [17]	48,M	Obesity	(+)	–	Low to High (Fluctuating)	AMS	–	–	Gradual improvement
6	A.M. Francesch et al. (2020) [17]	67,F	HTN, DM, CAD, Gout, Asthma	–	–	Normal to High	AMS	HTN		Gradual improvement
7	Yildiz Kaya et al. (2020) [18]	38,M	–	(+)	HCQ, Azithromycin, Osetalmivir, Oxygen	High levels for a few hours	Bilateral vision loss	–	Steroids	Recovered
8	J. Rogg et al. (2020) [19]	59,M	None	–	Ceftriaxone, Azithromycin, Remdesivir	High	AMS	–	–	Death
9	V. López Pérez et al. (2020) [20]	24,F	None	–	HCQ, Azithromycin, Ceftriaxone, Lopinavir/Ritonavir, LMWH	High	Hemiparesis, AMS, Hypoactive confusional syndrome with temporal-spatial disorientation	Pregnancy, tocilizumab	AEDs, LMWH, Steroids, Ceftriaxone	Improvement
10	Sripadma P.V. et al. (2020) [21]	25,F	–	(+)	HCQ, Oseltamivir, Piperacillin-Tazobactam, Azithromycin	High	Headache, Seizures, AMS	Pregnancy	–	Recovered
11	F.X. Doo et al. (2020) [22]	64,M	Smoking	(+)	HCQ, Azithromycin, Vancomycin, Ceftriaxone, Tocilizumab	–	Rhythmic jerking movements	Tocilizumab	AEDs, Midazolam, Lacosamide,	–
12	L. Ordonez-Boschetti et al. (2020) [23]	46,M	DM	(+)	HCQ, Azithromycin, Cefuroxime	High	AMS, Psychomotor agitation, Disconnection with the environment	–	–	Partial recovery with lower limb weakness
13	G. Conte et al. (2020) [24]	63, F	HTN	(+)	Lopinavir/Ritonavir, Piperacillin-Tazobactam, Anti-IL1	Initially high, then controlled	Gaze deviation, seizures	HTN	Diazepam, Lacosamide	Partial visual loss in the left eye
14	Talluri K et al. (2021) [25]	70,M	Asthma, HTN, CAD	–	Azithromycin, HCQ, Tocilizumab	High	Hyperactive delirium	HTN, Tocilizumab	Dexmedetomidine, Quetiapine, AEDs, Haloperidol	Death
15	Ketino Kobaidze et al. (2021) [26]	90, F	DM, HTN, DVT, Pulmonary embolism, Atrial flutter	–	–	High	AMS, Seizures	HTN	Labetalol, Lorazepam, AEDs	Recovered
16	Tissa Wijeratne et al. (2021) [27]	55,M	HTN, Obesity, CKD, OSA, Hypercholesterolaemia	–	Steroids	High	Lethargy, AMS	Smoker, HTN	–	Recovered
17	Sara Gómez-Enjuto et al. (2020) [28]	74,M	Multiple myeloma	–	HCQ, Lopinavir/Ritonavir, Ceftriaxone, Steroids, LMWH	High	Seizures, Blindness, Limb palsy	–	Diazepam, Lacosamide, AEDs, Verapamil	–
18	Fabiane Santos de Lima et al. (2021) [29]	43,F	Sickle cell disease	–	–	–	AMS, jerks, lethargy, seizures	–	Lorazepam, AEDs, Lacosamide	Recovered
19	Mohamed Elhassan et al. (2021) [30]	54,F	None	(+)	Amoxicillin/Clavulate, Clarithromycin	High	Seizures, Blindness	–	–	Gradual recovery but with impaired vision
20	Tarab Mansoor et al. (2021) [31]	31,F	Spina-bifida, scoliosis	–	–	Normal	Visual Changes, Headache	Reversible cerebral vasoconstriction syndrome	–	Recovered
21	Fabio Noro et al. (2021) [32]	67, F	Carotid endarterectomy with stenting, angioplasty	–	–	High	Seizures, AMS, loss of consciousness, agitation	–	–	Death
22	Ali Kerro (2021) [33]	85,M	AFib, DM, CKD, HTN	–	None	High	Behavioural Changes, AMS	HTN	Nicardipine, AEDs	Improvement
23	Ahmad J. Abdulsalam et al. (2021) [34]	46,M	Obesity, Smoking, HTN	(+)	Lopinavir/Ritonavir, HCQ	High	AMS, blurred vision, seizures	HTN	AEDs	Improvement
24	Louis Kishfy et al. (2020) [35]	58,M	Hyperlipidemia	(+)	Tocilizumab, HCQ, Azithromycin, Cefepime, Metronidazole	High	AMS	–	Nicardipine, Midazolam, Lorazepam	Improvement
25	Louis Kishfy et al. (2020) [35]	67,F	HTN, Obesity, DM	(+)	HCQ, Azithromycin, Ceftriaxone	High	AMS	HTN	Hydromorphone, Propofol, Midazolam, Lorazepam,	Improvement
26	Sarah C. Parauda et al. (2020) [36]	64,M	–	(+)	HCQ	High	Global aphasia	–	–	Inattentive, Right homonymous hemianopsia.
27	Sarah C. Parauda et al. (2020) [36]	73,M	–	(+)	HCQ	High	AMS	–	Benzodiazepines, Antipsychotics	Recovered
28	Sarah C. Parauda et al. (2020) [36]	65,F	HTN, DM	(+)	HCQ	High	Stuporous, repetitive blinking	HTN	–	Mild cognitive deficit with temporal disorientation
29	Sarah C. Parauda et al. (2020) [36]	74,F	Hypothyroidism, Hyperlipidemia, DM	(+)	HCQ, Tocilizumab	High	AMS with intermittent agitation, arm weakness	Tocilizumab	–	Improvement
30	F. D'Amore et al. (2020) [37]	64,F	–	–	–	–	AMS, reduced visual acuity	–	–	Improvement
31	Laura Llansó et al. (2020) [38]	66,F	–	–	Lopinavir/Ritonavir, HCQ, Azithromycin, Anti-IL1, Tocilizumab	–	AMS	Tocilizumab	Labetalol	Death
32	Antonio Colombo et al. (2021) [39]	54,M	–	(+)	Lopinavir/Ritonavir, Azithromycin, Ceftriaxone	High	Seizure	–	–	Improvement
33	Antonio Colombo et al. (2021) [39]	63,F	–	(+)	Lopinavir/Ritonavir, HCQ, Piperacillin/Tazobactam, LMWH, Anti-IL1	High	Seizures	–	Diazepam, AEDs	Recovered
34	Antonio Colombo et al. (2021) [39]	64,M	–	(+)	Lopinavir/Ritonavir, Piperacillin/Tazobactam	Normal	Seizures, tetraperesis	–	–	Recovered
35	Antonio Colombo et al. (2021) [39]	64,F	–	(+)	Lopnavir/Ritonavir, HCQ	High	AMS, unresponsive, blurred vision	–	–	Recovered
36	Antonio Colombo et al. (2021) [39]	68,M	–	(+)	Hydorxychloroquine, Lopinavir/Ritonavir	High	Visual disturbances, hypotonia	–	–	Recovered
37	Antonio Colombo et al. (2021) [39]	57,F	–	(+)	Levofloxacin, HCQ	High	Visual disturbance, seizures, aphasia, disinhibition, fatuity, visual hallucinations	–	Diazepam	Recovered
38	Abdenour Djellaoui et al. (2021) [40]	69,F	CAD, Endometrial cancer, Breast cancer	–	–	High	Seizures, mutism, AMS	–	–	Recovered
39	Ritwik Ghosh et al.(2020) [41]	33,F	None	–	Azithromycin, Steroids	–	Hallucinatory palinopsia	–	–	Recovered
40	Daniel Aguiar DIAS et al. (2020) [42]		–	(+)	–	–	AMS	–	–	–
41	Daniel Aguiar DIAS et al. (2020) [42]		–	(+)	–	–	AMS	–	–	–
42	Sofía Lallana et al. (2021) [43]	49,F	None	–	Lopinavir/Ritonavir, Chloroquine, Steroids, Tocilizumab	Normal	Paresis, Visual disturbance	Tocilizumab	Nimodipine	Focal sequel (paresis)
43	Sofía Lallana et al. (2021) [43]	36,F	None	–	Lopinavir/Ritonavir, Chloroquine, Steroids, Tocilizumab	Normal	Seizures, AMS, Paresis	Tocilizumab	AEDs	Focal sequel (paresis)
44	Sofía Lallana et al. (2021) [43]	66,M	None	–	Chloroquine, Corticosteroids	Normal	Status Epilepticus, AMS	–	AEDs	Remains guarded (stabilized, transferred to another acute hospital)
45	Sofía Lallana et al. (2021) [43]	53,M	None	–	Lopinavir/Ritonavir, Chloroquine, Tocilizumab, Convalescent plasma	Normal	Seizures, AMS	Tocilizumab	AEDs	Recovered
46	Sofía Lallana et al. (2021) [43]	55,F	HTN, Dyslipidemia, Obesity, CKD, DM, PV	–	Lopinavir/Ritonavir, Daruvir/Cobicistat, Chloroquine	Normal	Seizures	HTN	AEDs	Recovered
47	Sofía Lallana et al. (2021) [43]	70,M	DM, Liver transplant rejection	–	Steroids	High	Seizures, Paresis	–	AEDs	Death
48	Sofía Lallana et al. (2021) [43]	66,F	HTN, Dyslipidemia, DM, DVT	–	Lopinavir/Ritonavir, Chloroquine, Tocilizumab, Steroids, Convalescent plasma	High	Seizures, AMS	HTN, Tocilizumab	AEDs	Recovered
49	Sofía Lallana et al. (2021) [43]	68,M	HTN, Dyslipidemia, DM, OSA, Obesity	–	Steroids	High	Status Epilepticus, AMS	HTN	AEDs	Death
50	Alison M. Hixon et al. (2021) [44]	69,F	HTN, hyperlipidemia	(+)	Azithromycin, HCQ, Ceftriaxone, Steroids	High	Seizure-like jerking of left side, gaze deviation	HTN	Lorazepam, Hydralazine	Visual dysfunction
51	Alison M. Hixon et al. (2021) [44]	55,F	HTN, DM	(+)	HCQ, Steroids, Azithromycin, Ceftriaxone	High	Vision loss, vertigo, seizure, AMS	HTN	Lorazepam, AEDs	Recovered
52	Alison M. Hixon et al. (2021) [44]	65,M	HTN, DM, pyoderma gangrenosum	–	Steroids, Remdesivir, Azithromycin, Ceftriaxone	High	Seizures	HTN	AEDs	Recovered
53	Ornella Manara et al. (2021) [45]	56,M	None	–	HCQ, Steroids	High	AMS	–	AEDs	Improvement
54	Ornella Manara et al. (2021) [45]	21,M	Alport syndrome	(+)	HCQ, Steroids	–	AMS	–	–	Improvement
55	Hossein Mozhdehipanah et al. (2020) [46]	39,M	None	(+)	Cetriaxone, HCQ	Low	Seizures	Opium overdose	Labetalol, AEDs	Left side hemiparesis with improvement of symptoms
56	Alejandro Garcia Rodriguez et al. (2020) [47]	35,F	Hypothyroidism	–	–	High	Seizures, blindness	Pregnancy	AEDs, LMWH, Steroids, Ceftriaxone	Recovered

Abbreviations: PRES Posterior Reversible Encephalopathy Syndrome, M Male, F Female, HTN Hypertension, DM Diabetes Mellitus, OSA Obstructive Sleep Apnea, CKD Chronic Kidney Disease, GERD Gastroesophageal Reflux Disease, Afib Atrial Fibrillation, CAD Coronary Artery Disease, DVT Deep Vein Thrombosis, PV Peripheral Vasculopathy, HCQ hydroxychloroquine, Anti-IL1 Anti-Interleukin 1, LMWH Low Molecular Weight Heparin, SBP Systolic Blood Pressure, MAP Mean Arterial Pressure, DTR Deep Tendon Reflexes, GTC generalized tonic-clonic, AMS Altered Mental Status (including confusion, drowsiness, encephalopathy, delirium, disorientation and impaired conciousness), AEDs Anti-Epileptic Drugs, (−) data not reported.

**Table 2:** Diagnostic and Radiological findings of PRES in COVID-19 positive patients.

Patient No.	Author, Year	MRI Findings	Head CT Findings	Cerebrovascular Imaging
1	Lucia Princiotta Cariddi et al. (2020) [14]	T2/FLAIR: Vasogenic edema; GRE: with right temporal lobe hypodensity	Posterior frontal and temporo-parieto-occipital symmetric bilateral hypodensity of the subcortical WM Left occipital parenchymal hemorrhage	CTA: absence of vascular malformation, alterations of posterior circle vessel caliber (vasoconstriction mechanism)
2	Pria Anand et al. (2020) [15]	T2 WMH in parietal and occipital lobes; SWI with focus of susceptibility artifact in right frontal lobe	Posterior cortical paramedian hypodensity consistent with cerebral edema	CTV: Normal Vasculature
3	Pria Anand et al. (2020) [15]	T2 WMH involving right side of splenium of corpus callosum	Bilateral occipital hypodensities	None
4	Amit Agarwal et al. (2020) [16]	T2-FLAIR: areas of subcortical signal changes with edema in the occipital lobes. ADC: increased signal representing vasogenic edema	Unremarkable	–
5	A.M. Francesch et al. (2020) [17]	T1 FLAIR: vasogenic edema in the posterior parieto-occipital regions with blood. DWI: small infarct in the right occipital region SWI: diffuse petechial hemorrhages throughout the corpus callosum	Focal edema in the posterior parietooccipital regions bilaterally, subcortical in distribution, with a small right-sided hemorrhage.	CTV: Normal Vasculature
6	A.M. Francesch et al. (2020) [17]	DWI:Edema in posterior parieto-occipital lobe and right frontal lobe, basal ganglia, and cerebellar hemispheres. SWI: Extensive superimposed hemorrhages in the parieto-occipital region	Edema in the bilateral parietooccipital regions with associated mass effect and cortical sulcal effacement	MRA: Unremarkable
7	Yildiz Kaya et al. (2020) [18]	T2 FLAIR: bilateral occipital, frontal cortical and splenium of corpus callosum WMH DWI: Vasogenic edema	–	–
8	J. Rogg et al. (2020) [19]	FLAIR, DWI: Subcortical deep WMH with increased diffusivity. Hyperintense signal without restricted diffusion in the deep gray matter	Symmetric hypoattenuation of the posterior subcortical cerebral WM and external capsules	–
9	V. López Pérez et al. (2020) [20]	T2 FLAIR: Bilateral cortical and subcortical WMH (R > L)	Normal	CTA: normal
10	Sripadma P.V. et al. (2020) [21]	FLAIR: Bilateral parieto-occipital WMH DWI, ADC: Vasogenic Edema	Symmetrical parieto-occipital hypodensities with small hemorrhages bilaterally	CTA, CTV normal
11	F.X. Doo et al. (2020) [22]	FLAIR: Symmetric cerebral edema greatest in the parieto-occipital WM GRE: Multifocal foci of hemorrhage	Foci of hemorrhage with bilateral posterior cerebral vasogenic edema	MRA: Normal
12	L. Ordonez-Boschetti et al. (2020) [23]	T2-FLAIR: WMH bilaterally in the frontal, occipital lobes.	–	–
13	G. Conte et al. (2020) [24]	T2 FLAIR: WMH in the posterior hemisphere regions SWI: Subarachnoid blood effusion along the left precentral sulcus.	–	MRV: excluded intracranial thrombosis
14	Talluri K et al. (2021) [25]	FLAIR: Symmetric cortical and subcortical WMH bilaterally in frontotemporal lobes, occipital lobes and posterior thalami. DWI: Restricted diffusion in same regions	Symmetric abnormal low attenuation bilaterally in posterior occipital lobes involving the subcortical WM and the cortex.	–
15	Ketino Kobaidze et al. (2021) [26]	FLAIR: Subcortical and cortical WMH in the parietooccipital lobes (L > R) and the left temporal lobe.	Unremarkable	–
16	Tissa Wijeratne et al. (2021) [27]	T2 FLAIR: Bilateral parieto-occipital WMH. SWI: Cerebral microbleeds in the basal ganglia region.	Bilateral hypodensities around posterior parietal-occipital regions	
17	Sara Gómez-Enjuto et al. (2020) [28]	T2 FLAIR: Bilateral WMH predominantly in frontoparietal and occipital subcortical areas	Cortical and subcortical hypodensities, with predominance on the parietooccipital regions	–
18	Fabiane Santos de Lima et al. (2021) [29]	MRI: T2 FLAIR: WMH in the bilateral cerebral hemispheres; DWI: restricted diffusion in the right temporooccipito-parietal region.	–	–
19	Mohamed Elhassan et al. (2021) [30]	T1: Symmetric WMH in the bilateral occipital lobe cortex	Relative hypodensities posteriorly	–
20	Tarab Mansoor et al. (2021) [31]	T2-FLAIR: WMH in the parietooccipital the frontal lobes.	No signs of intracranial hemorrhage or ischemia	CTA: beading pattern mainly in the basilar artery, CTV: Unremarkable
21	Fabio Noro et al. (2021) [32]	–	Areas of bilateral parieto-occipital hypodensity	–
22	Ali Kerro (2021) [33]	FLAIR: Normal. DWI, ADC: Negative	Hypodensities in the right cerebellar hemisphere and bilateral subcortical occipital lobe	–
23	Ahmad J. Abdulsalam et al. (2021) [34]	T2 FLAIR: asymmetrical bilateral posterior parieto-occipital, centrum semi ovale and corona radiata WMH and features of vasogenic edema	Asymmetrical hypodensity within the bilateral parieto-occipital WM	‘-
24	Louis Kishfy et al. (2020) [35]	T2 FLAIR: subcortical WMH of occipital and posterior temporal lobes with effacement of adjacent sulci.	Characteristic convexal subarachnoid hemorhage	–
25	Louis Kishfy et al. (2020) [35]	T2 FLAIR: WMH of the right occipital lobe and the left cerebellar hemisphere with effacement of the adjacent local sulci. SWI: Petechial Hemorrhages		
26	Sarah C. Parauda et al. (2020) [36]	T2 FLAIR: Confluent bilateral WMH of occipital lobes, thalamus and internal capsule.	Bilateral occipital confluent WM hypodensities and patchy lucencies in the bilateral frontoparietal WM and posterior limb of the left internal capsule	–
27	Sarah C. Parauda et al. (2020)^36^	T2 FLAIR: Bilateral subcortical occipital WMH compatible with vasogenic edema	Confluent hypoattenuation in the bilateral parietooccipital WM	–
28	Sarah C. Parauda et al. (2020) [36]	T2 FLAIR: Mild subcortical bilateral occipital edema	Symmetric hypoattenuation involving the bilateral occipital subcortical WM	–
29	Sarah C. Parauda et al. (2020) [36]	T2 FLAIR: WMH in bilateral parieto-occipital lobes. DWI, SWI: Diffusion restriction and foci of susceptibility hypointensity within the areas of T2 hyperintensity	Hypoattenuation in the parietooccipital WM bilaterally	–
30	F. D'Amore et al. (2020) [37]	T2 FLAIR: WMH in the parieto-occipital lobes	Extensive bilateral subcortical edema with multifocal hemorrhages, predominantly in the occipital lobes	CTA: Unremarkable
31	Laura Llansó et al. (2020) [38]	–	Temporo-occipital WM hypodensity with symmetric obliteration of the sulci	–
32	Antonio Colombo et al. (2021) [39]	–	Areas of subcortical hypodensity in both occcipital regions, with greater expression on the left	–
33	Antonio Colombo et al. (2021) [39]	–	–	–
34	Antonio Colombo et al. (2021) [39]	FLAIR: Diffuse symmetrical WMH mainly affecting the occipital lobes bilaterally	–	–
35	Antonio Colombo et al. (2021) [39]	–	–	–
36	Antonio Colombo et al. (2021) [39]	–	–	–
37	Antonio Colombo et al. (2021) [39]	–	–	–
38	Abdenour Djellaoui et al. (2021) [40]	T2 FLAIR, ADC: Hyperintensities in temporo-occipital lobes; 3D T1 gadolinium sequence: Bilateral temporo-occipital leptomeningeal enhancement	–	Normal
39	Ritwik Ghosh et al.(2020) [41]	T2: WMH in bilateral occipital gray–white interface. FLAIR: Hyperintensities suggestive of vasogenic edema in bilateral occipital and frontal gray–white interface.	–	–
40	Daniel Aguiar DIAS et al. (2020) [42]	FLAIR: extensive bilateral parieto-occipital vasogenic edema; DWI: Diffusion restriction in some areas. SWI: Hypointensities	Bilateral parieto-occipital subcortical hypodensities associated with bilateral hemorrhages	MRA: Normal
41	Daniel Aguiar DIAS et al. (2020) [42]	–	Bilateral massive subcortical intra-parenchymal acute hematomas surrounded by edema	CTA: Normal
42	Sofía Lallana et al. (2021) [43]	–	Posterior hypodensities suggestive of vasogenic edema	CTA: proximal stenosis in both posterior cerebral arteries
43	Sofía Lallana et al. (2021) [43]	T2: Microbleeds; DWI, FLAIR: Asymmetric hemispheric involvement of the vasogenic edema.	–	–
44	Sofía Lallana et al. (2021) [43]	Symmetric parieto-occipital involvement	–	–
45	Sofía Lallana et al. (2021) [43]	Symmetric parieto-occipital involvement	–	–
46	Sofía Lallana et al. (2021) [43]	T2: Microbleeds in Parieto-Occipital regions	–	–
47	Sofía Lallana et al. (2021) [43]	Symmetric parieto-occipital involvement	–	–
48	Sofía Lallana et al. (2021) [43]	Symmetric parieto-occipital involvement	–	–
49	Sofía Lallana et al. (2021) [43]	T2 FLAIR, DWI: Asymmetric hemispheric involvement of the vasogenic edema.	–	–
50	Alison M. Hixon et al. (2021) [44]	T2 FLAIR: parietal and occipital cortex and subcortical WMH	–	–
51	Alison M. Hixon et al. (2021) [44]	T2 FLAIR: occipital, parietal, and posterior frontal WMH and edema	–	MRA, MRV: Normal
52	Alison M. Hixon et al. (2021) [44]	T2 FLAIR: WMH in the posterior frontal lobes and frontoparietal junctions; DWI: diffusion restriction at right parietal Lobe	Intraparenchymal hemorrhage in the right parietal lobe	–
53	Ornella Manara et al. (2021) [45]	T2 FLAIR: Corticosubcortical WMH in right frontal lobe, right posterior parietal lobe, left temporo-occipital region DWI: hyperintensity	Corticosubcortical mild hypodensity with swelling involving right frontal lobe, right posterior parietal lobe and left temporo-occipital region	–
54	Ornella Manara et al. (2021) [45]	T2 FLAIR, DWI, ADC: Symmetrical corticosubcortical WMH in temporo-occipital and in posterior and medial aspect of parietal lobe.	–	–
55	Hossein Mozhdehipanah et al. (2020) [46]	T2 FLAIR: Bilateral posterior parieto-occipital cortical and subcortical WMH extending to the frontal lobes.	Bilateral posterior parieto-occipital hypodensity in the cortical and subcortical WM extending to frontal lobes, and cortical hemorrhage in the right parietal lobe	–
56	Alejandro Garcia Rodriguez et al. (2020) [47]	–	–	–

Abbreviations: PRES Posterior Reversible Encephalopathy Syndrome, FLAIR Fluid-Attenuated Inversion Recovery, DWI Diffusion Weighted Imaging, SWI Susceptibility Weighted Imaging, ADC Apparent Diffusion Coefficient, GRE Gradient Echo Sequences, WM White Matter, WMH White Matter Hyperintensities, CTA CT Angiography, CTV CT Venography, MRA Magnetic Resonance Angiography, MRV Magnetic Resonance Venography, (−) data not reported.

The mean age of the patients was 56.6 ± 15.3 years (range 21–90 years). Highest number of cases were reported in the USA (n = 25; 44.6%) and Italy (n = 11; 19.6%). Out of 56 patients, 30 (53.6%) were females and 24 (42.9%) were males whereas the gender of two patients was not disclosed. Hypertension (n = 16; 28.6%) and diabetes mellitus (n = 13; 23.2%) were the most commonly reported comorbidities.

In terms of COVID-19 presentation, fever (n = 29, 51.8%), dyspnea (n = 24; 42.9%) and cough (n = 21; 37.5%) were the leading presenting complaints; and its management primarily included hydroxychloroquine/chloroquine (n = 31; 55.4%), azithromycin (n = 15; 26.8%), lopinavir/ritonavir (n = 15; 26.8%), corticosteroids (n = 14; 25.0%) and tocilizumab (n = 9; 16.1%). However, 29 critical patients (51.8%) also required mechanical ventilation. Blood pressure in 39 patients (69.6%) was reported to be higher than the normal recommended values and CRP levels were elevated in 14 patients (25.0%).

**Fig. 2:**
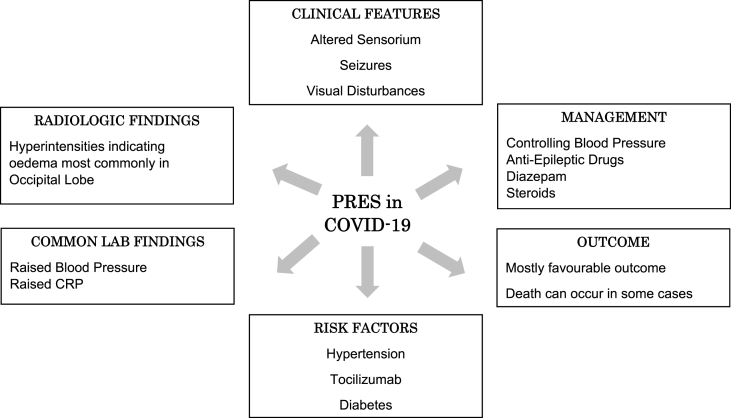
Summary of PRES in COVID-19.

**Fig. 3:**
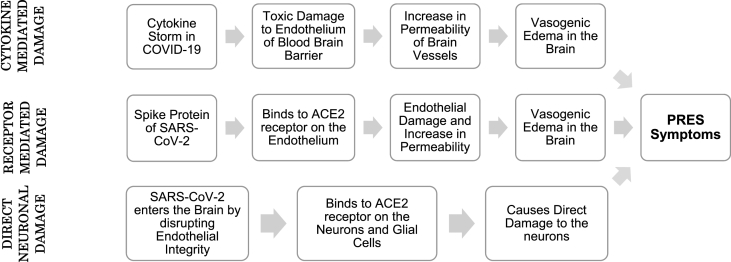
Models of pathogenesis of PRES in COVID-19.

The mean time for onset of PRES symptoms was 19.9 days after hospitalization, varying from a mean of 15.2 days in patients with no comorbidities and 23.0 days in patients with comorbidities. Patients most commonly presented with altered mental status (n = 33; 58.9%), seizures (n = 26, 46.4%) and visual disturbances (n = 13; 23.2%), with hypertension (n = 16; 28.6%) being the most notable risk factor. 51 patients (91.1%) had radiographic (MRI/CT) findings suggestive of PRES, whereas a clinical diagnosis was made in the remaining patients. 22 patients (39.3%) were treated with different Anti-Epileptic Drugs (AEDs), with levetiracetam (n = 12; 21.4%) and valproic acid (n = 7; 12.5%) being the most common choices. 24 patients (42.9%) were reported to have achieved full medical recovery whereas 20 (35.7%) recovered only partially. Additionally, seven patients (12.5%) died within 2–4 weeks of the onset of PRES, while the outcome of four patients (7.1%) was not reported. A pictorial summary of major findings in our study and the proposed pathogenesis of COVID-19-associated-PRES is presented in Fig. 2, Fig. 3.

## 4 Discussion

With the COVID-19 pandemic still raging in many parts of the world, newer neurological manifestations of this virus are unfolding before the medical fraternity. Posterior Reversible Encephalopathy Syndrome (PRES) is one such manifestation being increasingly described with SARS-CoV-2 [41]. PRES is considered to be significantly underreported because of its overlapping non-specific symptoms and limited awareness [50]. However, given the excessive rise in cases and the need for intensive care in almost half the patients diagnosed with PRES [51], the early recognition of this clinical association becomes of paramount importance. This systematic review serves to identify demographic characteristics and most common clinical and radiological features in patients diagnosed with COVID-19 related PRES. This will keep practicing physicians up-to-date regarding this novel manifestation of COVID-19 and in this process, enhance patient care and prognosis.

PRES usually presents with a multitude of neurological symptoms, which may be acute or subacute in onset [51]. These include seizures, both focal and generalized [52], and in the most severe forms, status epilepticus [53]. Seizures were also a common presentation (46.4%) of PRES in our systematic review, second only to altered mental status (58.9%). Taking into account the frequent involvement of occipital lobes, visual disturbances are also very frequently reported in PRES [54], a finding seen in 23.2% of cases in our review. Besides these, non-specific findings such as lethargy, headache and hypotonia were also observed. Moreover, although the previous literature describes a preponderance towards females [51], no gender predominance was noted in the studies included hereby. PRES also shows a predilection towards the posterior lobes, another finding corroborated by our results. This can be explained by the scarce sympathetic innervation of the vasculature in the posterior lobes, compared with the anterior lobes that receive innervation from the cervical ganglion [51]. This innervation contributes to the autoregulation of cerebral blood flow and thus the ability to maintain blood supply to the anterior lobes irrespective of any extracerebral blood vessel pathology.

Various models have been described in the literature to serve as a pathological basis of PRES. Given the established association of hypertension with PRES, the most commonly accepted pathophysiological model involves a failure of autoregulation at high blood pressures and subsequent hyperperfusion that results in vasogenic edema [55] in the posterior lobes of the brain. Another theory proposes a contrasting model that suggests endogenous or exogenous toxic damage to the vascular endothelium, leading to a breach in the integrity of the vessel wall and disruption in the blood-brain barrier. The resulting increase in the vessel's permeability causes the characteristic edema of PRES [51]. Although hypertension was reported as a risk factor in some cases (28.6%), a considerable majority did not suffer from hypertension. This suggests that the latter model (involving endothelial dysfunction) provides stronger evidence for COVID-19 related PRES. It is supported by a well-described mediator of COVID-19 disease -‘the cytokine storm syndrome’ [56] - which damages the capillary endothelium of the cerebral vasculature. Following a COVID-19 infection, there is a steep rise in T-cells and macrophages in the human body [19], both of which contribute to this pro-inflammatory cytokine storm that drives the endothelial dysfunction. In addition, the virus Spike protein S1 binds the Angiotensin-Converting Enzyme 2 (ACE2) receptor [57] on capillary endothelium directly, and causes injury to it, ultimately increasing its permeability. The potential of COVID-19 to bring about this permeability change might be one of the reasons behind its association with PRES.

In addition to the above hypotheses, the possibility of direct viral damage to the brain can not be ignored. The detection of SARS-CoV-2 in cerebrospinal fluid of neurological patients presenting with COVID-19 presented the first definitive evidence of its neurotropic potential [58]. Several routes of virion entry into the Central Nervous System (CNS) have been debated upon, the most important of them being that the virion disrupts endothelial integrity of the blood-brain barrier (BBB), and hence gains entry into the brain [59]. It is also important to note that neurons and glial cells express the ACE-2 receptors [60], making them a potential target of COVID-19. This suggests that the COVID-19 virus disrupts the BBB, enters the CNS, and is capable of causing direct neurological damage to neurons and glial cells.

Moreover, almost a quarter of the patients included in our study suffered from diabetes as comorbidity, suggesting that there might exist a link between the two pathologies. It has been found that ketoacidosis in uncontrolled diabetes produces serum pro-inflammatory cytokines that cause endothelial damage and dysfunction [61], thus contributing to the pathophysiology of PRES in much the same way as SARS-CoV-2. The pro-inflammatory cytokines (IL-6 and TNF-alpha) in diabetic ketoacidosis regulate the expression of vascular endothelial growth factor, ultimately increasing vascular permeability [62], which then causes the vasogenic cerebral edematous changes seen in PRES. Through their similar cytokine-mediated vascular assault, COVID-19 and diabetes possibly complement each other in developing PRES.

COVID-19 disease severity has been directly linked with increased serum levels of IL-6 and IL-10 [63], and despite the hypothesis that these cytokines result in the cytokine storm that in itself causes PRES, it is interesting to note that an IL-6 receptor inhibitor i.e. tocilizumab has been linked with an increased risk for PRES [64,65]. It has been proposed to possess endothelial modulation properties [66] that lead to increased vessel permeability. This is especially important considering that tocilizumab has as yet been considered a very safe drug to manage the cytokine storm associated with COVID-19 [67]. This systematic review noted that 16.1% of patients were either treated for COVID-19 with tocilizumab or had a history of treatment with it. Owing to the endothelial modulation properties of tocilizumab, it is likely that it contributed to the development of PRES in these patients. More studies are needed to better answer this question but the available literature suggests that this risk should be weighed against the potential benefit of tocilizumab in mitigating cytokine storm syndrome.

One-fifth of the patients developed intracerebral hemorrhages, ranging from petechiae to large hematomas. Hemorrhage is a well-known complication of PRES [68], owing to endothelial injury. However, it can be postulated that COVID-19 may substantially increase the risk of intracerebral hemorrhage, because of its own association with coagulopathy and Disseminated Intravascular Coagulation (DIC) [69]. As a byproduct of the endothelial injury inflicted by SARS-CoV-2, there are thrombotic changes that result in DIC and greater susceptibility to hemorrhages [70]. Hence, COVID-19 may decrease the threshold for intracranial hemorrhage, thus worsening the prognosis for PRES.

Knowing the pathogenesis of SARS-CoV-2, it is not unlikely that it predisposes the at-risk populations to PRES. It is thus appropriate to expect more cases of COVID-associated PRES to be reported in the future. Both radiologists and neurologists must anticipate the development of PRES in patients of COVID-19, especially the ones who have had a history of hypertension. Hypertension and COVID-19 may have an additive effect that decreases the hypertensive threshold for developing PRES in a clinical setting, and thus calls for stricter control of blood pressure in COVID-19 patients. Given the reversibility of PRES, timely diagnosis and initiation of appropriate treatment are of paramount importance to avoid any lasting neurological damage.

The authors would also like to acknowledge a few limitations while disseminating information through this systematic review. Firstly, large-scale observational studies reporting PRES in COVID-19 have not been conducted yet, so our review only included cases that have been published to date, hence bringing generalizability of results into question. Secondly, there is an inherent heterogeneity owing to the individual nature of every patient. Lastly, a possibility of publication bias also exists whereby rare diseases with unique associations and presentations are more likely to be reported and published.

## 5 Conclusions

COVID-19 related PRES presents with a constellation of clinical symptoms including altered mental status, visual disturbances and seizures. It is primarily diagnosed on MRI with preferential involvement of posterior lobes of the brain. In terms of pathogenesis, current literature backs the theory of cytokine storm mediated increase in vessel permeability but the possibility of direct viral damage owing to the neurotropic potential of this virus cannot be neglected. When COVID-19 is developed on a background of hypertension and diabetes, there is a possibility that all these factors play an additive role in the development of PRES. Interestingly, tocilizumab has also been proposed as a causative factor in PRES, but at present, there is little evidence to support this hypothesis. Clinicians, especially neurologists and radiologists must consider PRES as a probable diagnosis when encountering COVID-19 patients with suggestive clinical and radiographic findings. If treated promptly, most patients with COVID-19-associated-PRES either recover completely or with mild residual neurological deficits, so timely recognition of this clinical entity is the key to preventing complications like hemorrhage, epilepsy and visual loss.

## Sources of funding

This research did not receive any grant from funding agencies in the public, commercial, or not-for-profit sectors.

## Provenance and peer review

Not commissioned, externally peer-reviewed.

## Ethical approval

This is a systematic review and did not require ethical approval.

## Consent

This is a systematic review, where authors verified that proper consent was obtained from patients in all of the studies included.

## Author contributions

SI and AUR conceived the idea and MAR along with HF established a search strategy. MAR, HF and AA retrieved the articles, and screened them for relevancy. After selecting relevant articles, MZA and AN ran quality assessment on the included articles. Data was extracted by AUR, MZA and AN. HF and MER proofread the extracted data and matched it with articles to eliminate errors. AUR and AA then worked on the write up. MAR, MER and SI provided critical assistance in proof reading and editing of the write up. All the authors approved the final version of the article.

## Trial registration number

1. Name of the registry: Research Registry
2. Unique Identifying number or registration ID: reviewregistry1255
3. Hyperlink to your specific registration (must be publicly accessible and will be checked): https://www.researchregistry.com/browse-the-registry#registryofsystematicreviewsmeta-analyses/registryofsystematicreviewsmeta-analysesdetails/6191ff9c59030b001e971eb4/

## Guarantor

I, Aqeeb Ur Rehman, the corresponding author for this review accept my role as the Guarantor for this research.

## Declaration of competing interest

The authors declare no conflicts of interest.
