# Supplementary material for: The association of posterior reversible encephalopathy syndrome with COVID-19: A systematic review
Source: Ann Med Surg (Lond). 2021 Nov 20;72:103080. doi: 10.1016/j.amsu.2021.103080 (PMC8605817; doi:10.1016/j.amsu.2021.103080)
Supplement: Multimedia component 1 [file mmc1.docx]

You are viewing as a guest [Login](https://amstar.ca/index.php)


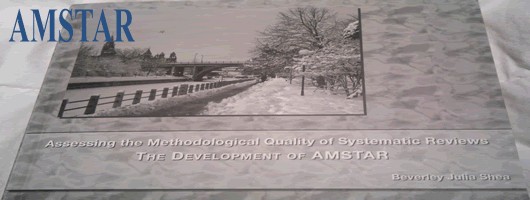


[Home](https://amstar.ca/index.php) [About Us](https://amstar.ca/About_Amstar.php) [Publications](https://amstar.ca/Publications.php) [Checklist](https://amstar.ca/Amstar_Checklist.php) [FAQs](https://amstar.ca/FAQs.php) [Contact Us](https://amstar.ca/contact_us.php)

# AMSTAR Checklist

[**Printer Friendly Version**](https://amstar.ca/docs/AMSTAR-2.pdf)

# Article Name:

The Association of Posterior Reversible Encephalopathy Syndrome with CO

1. **Did the research questions and inclusion criteria for the review include the components of PICO?**

For Yes: Optional (recommended)


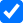
 Population
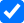
 Timeframe for follow up
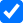
 Yes


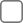

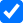
Intervention No


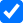
 Comparator group
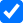
 Outcome

# Did the report of the review contain an explicit statement that the review methods were established prior to the conduct of the review and did the report justify any significant deviations from the protocol?

For Partial Yes:

The authors state that they had a written protocol or guide that included ALL the following:

For Yes:

As for partial yes, plus the protocol should be registered and should also have specified:


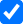
 review question(s)
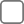
 a meta-analysis/synthesis plan, if appropriate, and


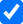
 a search strategy
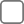
 a plan for investigating causes of heterogeneity


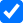
 inclusion/exclusion criteria
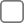
 a plan for investigating causes of heterogeneity


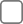
 Yes


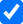
 Partial Yes
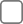
 No


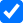
 a risk of bias assessment

# Did the review authors explain their selection of the study designs for inclusion in the review?

For Yes, the review should satisfy ONE of the following:


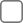
 Explanation for including only RCTs
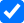
 Yes


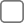

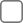
OR Explanation for including only NRSI No


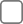
 OR Explanation for including both RCTs and NRSI

# Did the review authors use a comprehensive literature search strategy?

For Partial Yes (all the following): For Yes, should also have (all the following):


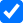
 searched at least 2 databases (relevant to research question)


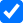
 provided key word and/or search strategy


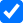
 justified publication restrictions (e.g. language)


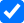
 searched the reference lists / bibliographies of included studies


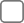
 searched trial/study registries


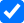
 included/consulted content experts in the field


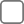
 where relevant, searched for grey literature


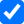
 conducted search within 24 months of completion of the review


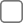
 Yes


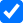
 Partial Yes
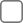
 No

# Did the review authors perform study selection in duplicate?

For Yes, either ONE of the following:


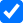
 at least two reviewers independently agreed on selection of eligible studies and achieved consensus on which studies to include


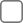
 OR two reviewers selected a sample of eligible studies and achieved good agreement (at least 80 percent), with the remainder selected by one reviewer.

# Did the review authors perform data extraction in duplicate?

For Yes, either ONE of the following:


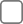
 at least two reviewers achieved consensus on which data to extract from included studies


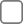
 OR two reviewers extracted data from a sample of eligible studies and achieved good agreement (at least 80 percent), with the remainder extracted by one reviewer.


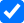
 Yes
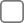
 No


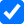
 Yes
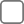
 No

# Did the review authors provide a list of excluded studies and justify the exclusions?

For Partial Yes: For Yes, must also have:


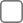
 provided a list of all potentially relevant studies that were read in full-text form but excluded from the review


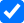
 Justified the exclusion from the review of each potentially relevant study


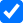
 Yes


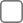
 Partial Yes
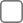
 No

# Did the review authors describe the included studies in adequate detail?

For Partial Yes (ALL the following): For Yes, should also have ALL the following:


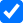
 described populations
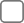
 described population in detail
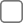
 Yes


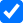
 described interventions
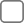
 described intervention in detail (including doses where relevant)


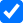
 described comparators
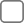
 described comparator in detail (including doses where relevant)


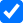
 described outcomes
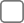
 described study’s setting


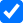
 described research designs
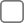
 timeframe for follow-up


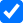
 Partial Yes
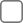
 No

# Did the review authors use a satisfactory technique for assessing the risk of bias (RoB) in individual studies that were included in the review?

**RCTs**

For Partial Yes, must have assessed RoB from

For Yes, must also have assessed RoB from:


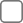

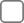
unconcealed allocation, and allocation sequence that was not truly random, and

Yes


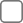

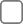

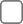
Partial Yes No


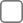
 lack of blinding of patients and assessors when assessing outcomes (unnecessary for objective outcomes such as all-cause mortality)

# NRSI


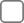
 selection of the reported result from

among multiple measurements or analyses of a specified outcome


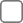
 Includes only NRSI

For Partial Yes, must have assessed RoB: For Yes, must also have assessed RoB:
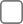
 from confounding, and
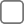
 methods used to ascertain exposures

and outcomes, and


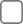
 from selection bias
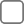
 selection of the reported result from

among multiple measurements or analyses of a specified outcome


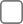
 Yes


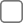
 Partial Yes
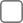
 No


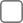
 Includes only RCTs

# Did the review authors report on the sources of funding for the studies included in the review?

For Yes


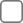
 Must have reported on the sources of funding for individual studies included in the review. Note: Reporting that the reviewers looked for this information but it was not reported by study authors also qualifies


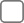
 Yes
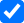
 No

# If meta-analysis was performed did the review authors use appropriate methods for statistical combination of results?

**RCTs**

For Yes:


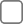
 The authors justified combining the data in a meta-analysis
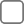
 Yes


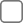
 AND they used an appropriate weighted technique to combine study results and adjusted for heterogeneity if present.


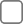
 AND investigated the causes of any heterogeneity


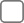
 No


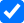
 No meta-analysis conducted

# For NRSI

For Yes:


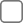
 The authors justified combining the data in a meta-analysis
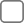
 Yes


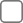
 AND they used an appropriate weighted technique to combine study results, adjusting for heterogeneity if present


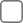
 AND they statistically combined effect estimates from NRSI that were adjusted for confounding, rather than combining raw data, or justified combining raw data when

adjusted effect estimates were not available


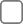
 AND they reported separate summary estimates for RCTs and NRSI separately when both were included in the review


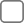
 No


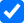
 No meta-analysis conducted

# If meta-analysis was performed, did the review authors assess the potential impact of RoB in individual studies on the results of the meta-analysis or other evidence synthesis?

For Yes:


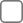
 included only low risk of bias RCTs
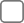
 Yes


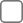
 OR, if the pooled estimate was based on RCTs and/or NRSI at variable RoB, the

authors performed analyses to investigate possible impact of RoB on summary estimates of effect.


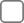
 No


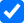
 No meta-analysis conducted

# Did the review authors account for RoB in individual studies when interpreting/ discussing the results of the review?

For Yes:


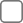

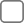
included only low risk of bias RCTs Yes


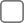
 No

OR, if RCTs with moderate or high RoB, or NRSI were included the review provided a discussion of the likely impact of RoB on the results

# Did the review authors provide a satisfactory explanation for, and discussion of, any heterogeneity observed in the results of the review?

For Yes:

There was no significant heterogeneity in the results Yes No

OR if heterogeneity was present the authors performed an investigation of sources of any heterogeneity in the results and discussed the impact of this on the results of the review

# If they performed quantitative synthesis did the review authors carry out an adequate investigation of publication bias (small study bias) and discuss its likely impact on the results of the review?

For Yes:

performed graphical or statistical tests for publication bias and discussed the likelihood and magnitude of impact of publication bias

Yes No

No meta-analysis conducted

# Did the review authors report any potential sources of conflict of interest, including any funding they received for conducting the review?

For Yes:

The authors reported no competing interests OR Yes

The authors described their funding sources and how they managed potential conflicts No of interest

To cite this tool: Shea BJ, Reeves BC, Wells G, Thuku M, Hamel C, Moran J, Moher D, Tugwell P, Welch V, Kristjansson E, Henry DA. AMSTAR 2: a critical appraisal tool for systematic reviews that include randomised or non-randomised studies of healthcare interventions, or both. BMJ. 2017 Sep 21;358:j4008.

Assess Article

Copyright © 2021 AMSTAR All Rights Reserved
